# Low levels of viral suppression among refugees and host nationals accessing antiretroviral therapy in a Kenyan refugee camp

**DOI:** 10.1186/s13031-017-0111-3

**Published:** 2017-06-02

**Authors:** Joshua B. Mendelsohn, Paul Spiegel, Alison Grant, Sathyanarayanan Doraiswamy, Marian Schilperoord, Natasha Larke, John Wagacha Burton, Jully A. Okonji, Clement Zeh, Bosco Muhindo, Ibrahim M. Mohammed, Irene N. Mukui, Njogu Patterson, Egbert Sondorp, David A. Ross

**Affiliations:** 1 College of Health Professions, Pace University, New York, NY USA; 2 MRC Tropical Epidemiology Group, Department of Infectious Disease Epidemiology, London School of Hygiene and Tropical Medicine, London, United Kingdom; 3 Public Health and HIV Unit, United Nations High Commissioner for Refugees, Geneva, Switzerland; 4 Department of Clinical Research, London School of Hygiene and Tropical Medicine, London, United Kingdom; 5 East Africa Regional Office, United Nations High Commissioner for Refugees, Nairobi, Kenya; 6 Center for Global Health Research and HIV-Research Laboratory, Kenya Medical Research Institute, Kisumu, Kenya; 7 National AIDS/STD Control Program, Nairobi, Kenya; 8 Department of Global Health and Development, London School of Hygiene and Tropical Medicine, London, United Kingdom; 9 World Health Organization, Geneva, Switzerland; 10 Center for Refugee and Disaster Response, Johns Hopkins Bloomberg School of Public Health, Baltimore, USA; 11 Clinical and Laboratory Standards Institute, Centers for Disease Control and Prevention Program-Kenya, Kisumu, Kenya

**Keywords:** Kenya, Refugee, Migration, Antiretroviral therapy, Adherence, HIV

## Abstract

**Background:**

Refugees and host nationals who accessed antiretroviral therapy (ART) in a remote refugee camp in Kakuma, Kenya (2011–2013) were compared on outcome measures that included viral suppression and adherence to ART.

**Methods:**

This study used a repeated cross-sectional design (*Round One* and *Round Two*). All adults (≥18 years) receiving care from the refugee camp clinic and taking antiretroviral therapy (ART) for ≥30 days were invited to participate. Adherence was measured by self-report and monthly pharmacy refills. Whole blood was measured on dried blood spots. HIV-1 RNA was quantified and treatment failures were submitted for drug resistance testing. A remedial intervention was implemented in response to baseline testing. The primary outcome was viral load <5000 copies/mL. The two study rounds took place in 2011-2013.

**Results:**

Among eligible adults, 86% (73/85) of refugees and 84% (86/102) of Kenyan host nationals participated in the *Round One* survey; 60% (44/73) and 58% (50/86) of *Round One* participants were recruited for *Round Two* follow-up viral load testing. In *Round One*, refugees were older than host nationals (median age 36 years, interquartile range, IQR 31, 41 vs 32 years, IQR 27, 38); the groups had similar time on ART (median 147 weeks, IQR 38, 64 vs 139 weeks, IQR 39, 225). There was weak evidence for a difference in the proportion of refugees and host nationals who were virologically suppressed (<5000 copies/mL) after 25 weeks on ART (58% vs 43%, *p* = 0.10) and no difference in the proportions suppressed at *Round Two* (74% vs 70%, *p* = 0.66). Mean adherence within each group in *Round One* was similar. Refugee status was not associated with viral suppression in multivariable analysis (adjusted odds ratio: 1.69, 95% CI 0.79, 3.57; *p* = 0.17). Among those not suppressed at either timepoint, 69% (9/13) exhibited resistance mutations.

**Conclusions:**

Virologic outcomes among refugees and host nationals were similar but unacceptably low. Slight improvements were observed after a remedial intervention. Virologic monitoring was important for identifying an underperforming ART program in a remote facility that serves refugees alongside host nationals. This work highlights the importance of careful laboratory monitoring of vulnerable populations accessing ART in remote settings.

**Electronic supplementary material:**

The online version of this article (doi:10.1186/s13031-017-0111-3) contains supplementary material, which is available to authorized users.

## Background

Life-long antiretroviral therapy (ART) that requires consistent access to medications and supportive services is a challenge in conflict-affected settings. By the end of 2013, 172 million people were affected by violent conflict worldwide, while 51.2 million people were forcibly displaced as a result of conflict, persecution, generalized violence, and human rights violations [1, 2]. Of these, 16.7 million were classified as refugees who had crossed an international border to escape a well-founded fear of persecution in the absence of protection in their home country. International humanitarian and refugee law suggest that refugees should receive an equivalent standard of medical care to host nationals in countries where they have sought protection [3]. In many settings, refugees and host nationals receive HIV services from the same facilities. An equivalent standard of HIV care should result in similar treatment outcomes. Few studies, however, have sought to rigorously compare HIV treatment outcomes between refugee and host national groups. Previous work has shown high levels of adherence to ART, increases in CD4 counts, and increases in survival among confict-affected populations [4, 5]. In a study comparing urban refugees situated in Kuala Lumpur with Malaysian host nationals, we found comparable levels of viral suppression [6]. Refugees, however, live in a variety of settings (e.g., camps, cities, etc.), and it is not known how these different environments might influence their HIV treatment outcomes [7]. Better data will help governments and humanitarian agencies improve ART delivery, while retaining more people along the HIV cascade of care [8]. We report on a repeated cross-sectional study of adherence and viral suppression among refugees and host nationals accessing ART in Kakuma Refugee Camp, Kenya, pre- and post- a remedial intervention that responded to poor baseline levels of viral suppression. Our aim was to compare viral suppression and adherence to ART among refugees and host nationals and to identify factors associated with viral suppression.

## Methods

### Study setting

Kakuma Refugee Camp is situated in a remote part of Northwestern Kenya. In 2011, the camp's Comprehensive Care Clinic was managed by the International Rescue Committee (IRC) and was overseen by the United Nations High Commissioner for Refugees (UNHCR). At study initiation (February 2011), the camp population was 82,409 (Somalia, 54%; Sudan, 32%; Ethiopia, 8%; Democratic Republic of the Congo, 5%). Local host nationals were primarily Turkana, a nomadic-pastoralist group. Adult HIV prevalence within the camp was 1.2% [9]. Clients who tested positive for HIV were counseled and started on multivitamins and cotrimoxazole, followed by ART at CD4 < 250 cells/ul. After six months on ART, clinic appointments were scheduled every six months; however, clients typically presented at the clinic pharmacy on a monthly basis to refill their ART prescriptions. At this time, they could request a counseling session with a nurse or community support worker. First-line regimens consisted of two nucleoside reverse transcriptase inhibitors (NRTIs), usually lamivudine with zidovudine, stavudine or tenofovir, combined with a non-nucleoside reverse transcriptase inhibitor (NNRTI) such as nevirapine or efavirenz, given twice daily. Six months prior to the study, stavudine was phased out as per 2009 World Health Organization (WHO) recommendations. Routine CD4-testing, counseling services, and nutritional supplementation including additional rations of cornmeal and peanut-based nutritional spread were provided to ART clients.

### Study design

This study used a repeated cross-sectional design with baseline (*Round One*) and follow-up (*Round Two*) segments. We initially designed the baseline cross-sectional survey. *Round Two* was initiated on the basis of *Round One* results.

#### Round One

A five-week cross-sectional survey was implemented between February and April 2011. All adults were invited to participate if they accessed their treatment from the Comprehensive Care Clinic, were ≥18 years old, had been taking ART for ≥30 days, had not missed ≥6 consecutive monthly pharmacy refills, and were not exclusively on ART for prevention of mother-to-child transmission. Recruitment occurred during regular clinic appointments or through an active recruitment protocol implemented by community health workers. Three attempts were made to contact all eligible adults by telephone or home visit.

#### Round Two

In response to very low proportions of participants who were virally suppressed in *Round One*, a remedial intervention was implemented by IRC in December 2011 that aimed to improve elements of the ART program. Intervention measures included hiring an additional clinical officer and a full-time adherence support counselor, construction of an additional adherence counseling room, pill counting during appointments, closer monitoring of the ART surveillance database, increased frequency of CD4 monitoring (once per year increased to twice per year), additional training for peer support counselors, additional adherence counseling during appointments, and intensified monitoring of clients with viral loads ≥5000 copies/mL. Baseline participants were re-contacted between September 2012 and June 2013 and invited to provide a *Round Two* follow-up viral load. Survey questionnaires were not completed in *Round Two*. Following Kenyan national guidelines, clients with a second consecutive viral load of ≥5000 copies/mL were switched to second-line ART.

#### Data sources and measures

A structured questionnaire was administered during *Round One*. Data was collected on a pen and paper survey administered in a face-to-face interview conducted in the respondent’s language of choice (usually the mother tongue). Prior to pre-testing, the questionnaire was translated from English into Swahili, Nga’turkana, Somali, French and Juba Arabic, and back-translated into English by an independent translator. Both versions were then reconciled. HIV RNA-1 concentrations were measured using dried blood spots collected on Whatman 903 filter paper and stored at -20 °C in the refugee camp pharmacy storage facility. Samples were shipped on dry ice to collaborating Kenya Medical Research Institute (KEMRI) laboratories. Self-reported adherence was measured using a 4-day dose-by-dose recall and a one-month recall visual analogue scale where participants were asked to mark an “X” representing the amount of medication they had taken during the previous month at any point along a 10 cm line [10, 11]. Adherence to pharmacy prescription refill schedule was estimated from pharmacy records as the proportion of refills collected divided by the total number of refills prescribed in the 24 months prior to the baseline interview or since the month of ART initiation, whichever was longer. The primary outcome was viral load <5000 copies/mL, consistent with WHO recommendations on minimum cut-offs for viral loads measured from dried spots [12].

#### Laboratory procedures

Viral loads were analyzed on the COBAS Ampliprep/Taqman platform (Roche Diagnostics Systems, Branchburg, New Jersey, USA). *Round One* samples from clients with two consecutive viral loads ≥5000 copies/mL were submitted for drug resistance testing. Sequencing was performed using a WHO-accredited in-house genotyping assay. Sequenced samples were analyzed on an ABI Prism 3130xl Genetic Analyzer (Applied Biosystems, Foster City, CA, USA) and edited using RECall v2.0 Software, using HIV-1 HXB2 as the reference sequence [13]. Protease (PR) and reverse transcriptase (RT) fragments were confirmed against the Stanford University HIV Drug Resistance Database Version 7.0 (http://hivdb.stanford.edu/) and the International AIDS Society Mutation List [14, 15].

### Statistical methods

Medians, proportions and corresponding 95% confidence intervals (95% CI) were used to compare outcomes. Risk factors were evaluated using unconditional logistic regression. Effect estimates included odds ratios (OR) and corresponding 95% CIs. A three-level hierarchical, step-wise modelling approach was used to order the entry of factors in multivariable models. Social action theory, which emphasizes the influence of social interaction and environmental structures on problem-solving activities affecting health behaviors [16], was selected as the conceptual framework given its consideration of socio-structural factors relevant for refugee camp settings. Drawing on social action theory, factors were grouped into levels representing “treatment contexts” i.e., socio-demographic factors; “self-change factors” i.e., knowledge measures; and “action state factors” i.e., adherence measures (see Additional files 1, 2 and 3). Associations with viral suppression were evaluated in univariable analyses using log-likelihood ratio tests. The “treatment context model” was fitted by adjusting for factors with *p* < 0.2 in univariable analyses. The “self-change model” was fitted by adjusting for all retained factors from the prior level, then adjusting again for any additional self-change factors meeting the *p* < 0.2 threshold. Given detected collinearities, the “action state model” was adjusted by factors from previous model levels, but not by additional factors within this level. The final multivariable model sequentially excluded factors with the highest *p*-values, until all factors met a *p* < 0.05 cut-off. Refugee status, age, and time on ART were retained throughout modelling as *a priori* covariates of interest. Given that adjustment by mediating factors can introduce over-adjustment bias, adherence factors were not included in the final model [17].

## Results

### Study population

**Fig. 1:**
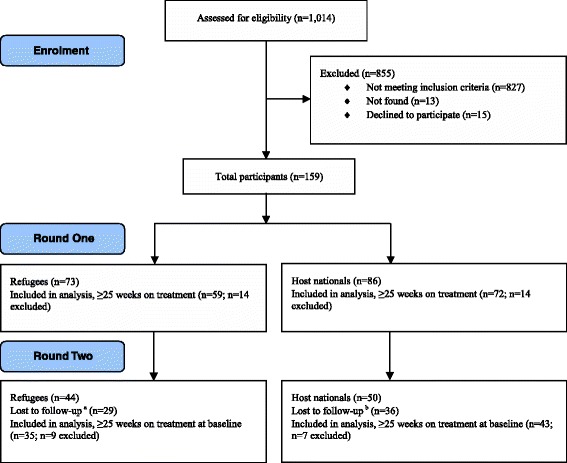
Flow diagram of study participation. Legend: ^a^Reasons for loss to follow-up included (refugees): 18% (13/73) were active clients but not found within the follow-up period; 12% (9/73) had missed ≥1 most recent pharmacy refills and were not found; 7% (5/73) of refugees were resettled to a different country; 1% (1/73) had died; and 1% (1/73) had transferred to a different clinic. ^b^Reasons for loss to follow-up included (host nationals): 19% (16/86) had missed ≥1 most recent pharmacy refills and were not found; 14% (12/86) were active clients but not found within the follow-up period; 5% (4/86) had transferred to a different clinic; 4% (3/86) had died; and 1% (1/86) were approached but declined

Among eligible HIV-positive clients who were enrolled in the ART program, 86% (73/85) of refugees and 84% (86/102) of Kenyan host nationals participated in *Round One*. Among refugees, 11% (9/85) declined and 4% (3/85) were not found; among host nationals 6% (6/102) declined and 10% (10/102) were not found (Fig. 1). Viral loads were collected from 159 clients during *Round One*, 83% (131/159) of whom were on treatment for ≥25 weeks and included in analyses. Refugees reported a median time in the host country of 9.8 years (IQR 4.5, 15.7), and median time since registration with UNHCR of 8.5 years (IQR 2.8, 14.9). HIV-positive adults from the refugee and host community had similar proportions of women (67% vs 66%, *p* = 0.91) while having comparable median durations on ART (147 vs 139 weeks, *p* = 0.65). Refugees were older than host nationals (median age 36 years vs 32 years, *p* = 0.02) (Table 1).

**Table 1:** Socio-demographic characteristics of refugees and host nationals

Factor	Round One, all			Round One, ≥25 weeks on treatment			Round Two			Not included in Round Two		
	Refugee (*n* = 73)	Host (*n* = 86)	*p*-value	Refugee (*n* = 59)	Host (*n* = 72)	*p*-value	Refugee (*n* = 35)	Host (*n* = 43)	*p*-value	Refugee (*n* = 29)	Host (*n* = 36)	*p*-value
Age, median yrs (IQR)	36 (31, 41)	32 (27, 38)	0.02^a^	37 (33, 43)	32 (27, 38)	0.001^a^	36 (33, 41)	28 (24, 38)	0.02^a^	39 (34, 43)	31 (27, 39)	0.04^a^
Female gender	49 (67)	57 (66)	0.91	38 (64)	48 (67)	0.79	21 (60)	27 (63)	0.80	17 (71)	2 (72)	0.90
No earned income^c^	63 (86)	70 (81)	0.41	49 (83)	57 (79)	0.57	28 (80)	33 (78)	0.73	21 (88)	24 (83)	0.63
Married/cohabiting	29 (40)	39 (45)	0.48	23 (39)	32 (44)	0.53	15 (43)	24 (56)	0.26	8 (33)	8 (28)	0.65
Nationality												
Kenyan	0 (0)	86 (100)	<0.001^b^	0 (0)	72 (100)	<0.001^b^	0 (0)	43 (100)	<0.001^b^	0 (0)	29 (100)	<0.001^b^
Somali, Ethiopian, Eritrean^d^	36 (49)	0 (0)		29 (49)	0 (0)		14 (40)	0 (0)		15 (63)	0 (0)	
Sudanese	20 (27)	0 (0)		18 (31)	0 (0)		14 (18)	0 (0)		4 (17)	0 (0)	
Rwandese, Congolese, Burundian	17 (23)	0 (0)		12 (20)	0 (0)		7 (20)	0 (0)		5 (21)	0 (0)	
Travel for ≥1 continuous month in past year	8 (11)	22 (26)	0.02	7 (12)	20 (28)	0.03	3 (9)	8 (19)	0.21	4 (17)	12 (41)	0.05
Self-reported consistent adherence to medication schedule	51 (71)	72 (84)	0.05	44 (76)	60 (83)	0.29	26 (74)	38 (88)	0.11	18 (78)	22 (76)	0.84
Incorrect ART dosing	26 (36)	11 (13)	0.001	20 (34)	8 (11)	0.002	13 (37)	4 (9)	0.003	7 (29)	4 (14)	0.17
Time on ART, median wks (IQR)	147 (38, 264)	139 (39, 225)	0.65^a^	202 (86, 270)	165 (96, 240)	0.25^a^	192 (80, 268)	190 (120, 235)	0.59^a^	208 (87, 275)	120 (56, 242)	0.29^a^
Time from HIV diagnosis to ART, median wks (IQR)	8 (0, 44)	15 (1, 56)	0.26^a^	4 (0, 23)	9 (0, 52)	0.11^a^	1 (0, 13)	9 (0, 56)	0.04^a^	10 (0, 67)	13 (1, 47)	0.86^a^
Time in host country, median wks (IQR)	507 (234, 814)	–	–	525 (265, 886)	–	–	596 (309, 948)	–	–	506 (234, 809)	–	–

Values are numbers (%) unless otherwise stated; *p-*values are chi-square tests unless otherwise stated; *IQR* interquartile range

^a^Mann-Whitney two-sample statistic (Wilcoxon rank-sum test)

^b^Fisher’s exact test

^c^Not including financial assistance provided within refugee camp

^d^Somalis, Ethiopians, and Eritreans were grouped as Somalis often reported Ethiopian nationality to conceal identities

### Round One

#### Virologic outcomes and adherence

Of all clients on treatment for ≥25 weeks, 50% (65/131) had viral loads <5000 copies/mL. There was weak evidence that a higher proportion of refugees were suppressed at this level when compared with host nationals (58% vs 43%; *p* = 0.10). There was strong evidence, however, that refugees had a lower median viral load at baseline (3,830 copies/mL vs 7,905; *p* = 0.03) (Table 2). Mean adherence to pharmacy refill schedule was 95.6% (95%CI: 91.6, 99.6) for refugees, compared with 90.7% (95%CI 91.6, 99.6) for host nationals (*p* = 0.11) (Additional file 4). Comparing refugees and host nationals on adherence measured by 4-day self-report (94.2% vs 92.2%, *p* = 0.62) and 1-month visual analogue scale (90.8% vs 87.3%, *p* = 0.26) revealed only minor differences between groups.

**Table 2:** Comparison of HIV viral loads among refugees and host nationals taking ART for ≥25 weeks

Viral load	Group	Total, N (100%)	<5000 copies/mL, n (%)	*p*-value	Median, copies/mL (IQR)	*p*-value
Round One	Refugee	59	34 (58)	0.10	3830 (1460, 14800)	0.03^a^
	Kenyan	72	31 (43)		7905 (2625, 38900)	
	All	131	65 (50)		5010 (1920, 22400)	
Round Two	Refugee	35	26 (74)	0.66	1311 (<400, 6361)	0.41^a^
	Kenyan	43	30 (70)		1411 (<400, 28107)	
	All	78	56 (72)		1368 (<400, 7477)	
Differences in median viral load, baseline v. follow-up ^b^: refugee, *p* = 0.04; Kenyan, *p* = 0.03; All, *p* = 0.002						

*p*-values are chi-square tests unless otherwise stated; *IQR* interquartile range

^a^Mann-Whitney two-sample statistic (Wilcoxon rank-sum test)

^b^Wilcoxon signed-rank test

#### Factors associated with virologic outcomes

Within the “treatment contexts level” (Additional file 1), ≥48 weeks between HIV diagnosis and ART start was associated with a fourfold increase in the odds of viral suppression (AOR: 3.98, 95% CI 1.44, 11.01; *p-trend* = 0.006). The weak association between refugee status and viral suppression in crude analyses (OR: 1.88, 95% CI 0.93, 3.81; *p* = 0.08) was further attenuated after adjusting for age group, time on ART, time from HIV diagnosis to ART start, place of ART start, refill difficulties, and food security (AOR: 0.51, 95% CI 0.08, 3.18; *p* = 0.46). No evidence was found for associations with “self-change factors” (Additional file 2). Among exposures in the “action state” model, self-reported adherence to medication schedule (AOR: 2.80, 95% CI 0.99, 7.90; *p* = 0.04) and self-reported dosing schedule (i.e., correct reporting of dosing schedule) were associated with viral suppression (AOR: 3.33, 95% CI 1.20, 9.24; *p* = 0.02) (Additional file 3). There was also evidence that self-reported adherence over the past month was associated with decreased odds of viral suppression (AOR = 0.71, 95% CI 0.44, 1.14; *p* = 0.05). This association may have been confounded by adjustment for refill difficulties in the past three months. The magnitude of this confounding was 58% (OR – AOR/AOR).

In the final multivariable model (Table 3), longer duration between HIV diagnosis and ART start (≥48 weeks, AOR: 3.61, 95% CI 1.37, 9.47; *p-trend* = 0.01), self-reported adherence to medication schedule (AOR: 3.12, 95% CI 1.14, 8.49; *p* = 0.02), and self-reported dosing schedule (AOR: 2.52, 95% CI 0.96, 6.58; *p* = 0.05), were strongly associated with viral suppression. There was no evidence for an association between refugee status and viral suppression (AOR: 1.69, 95% CI 0.79, 3.57; *p* = 0.17). Age and time on ART were not associated with the outcome in any models. All effects in the final model were adjusted for age group, refugee status, time on ART, and time from HIV diagnosis to ART start.

**Table 3:** Final multivariable model showing factors associated with viral suppression among refugees and host nationals on ART for ≥25 weeks at baseline in Kakuma, Kenya (*N* = 128^a^)

Factor	Prevalence <5000 copies/mL, n/N (%)	Crude odds ratio (95% CI)	*p*-value	Adjusted odds ratio (95% CI)^b^	*p*-value
Age group (years)^c^					
18+	14/31 (45)	1	*p(tr)* = 0.90	1	*p(tr)* = 0.82
30+	33/63 (52)	1.34 (0.56, 3.17)		1.47 (0.57, 3.76)	
40+	16/34 (47)	1.08 (0.41, 2.87)		0.90 (0.31, 2.59)	
Refugee status					
Host	9/20 (45)	1	*p* = 0.08	1	*p* = 0.17
Refugee	19/41 (46)	1.88 (0.93, 3.81)		1.69 (0.79, 3.57)	
Time on ART (years)^c^	35/67 (52)				
0-		1	*p(tr)* = 0.49	1	*p(tr)* = 0.76
1-	30/71 (42)	1.06 (0.36, 3.09)		0.80 (0.26, 2.49)	
2+	33/57 (58)	1.34 (0.49, 3.64)		1.00 (0.34, 2.95)	
Time from HIV diagnosis to ART start (weeks)^c^					
0-	8/29 (28)	1	*p(tr)* = 0.006	1	*p(tr)* = 0.01
24-	6/13 (46)	2.25 (0.58, 8.78)		2.71 (0.66, 11.11)	
48+	49/86 (57)	3.48 (1.39, 8.72)		3.61 (1.37, 9.47)	
Adherence to medication schedule, self-reported^b^					
Inconsistent	7/25 (28)	1	*p* = 0.02	1	*p* = 0.02
Consistent	56/103 (54)	3.06 (1.18, 7.96)		3.12 (1.14, 8.49)	
Self-reported dosing schedule^b, d^					
Incorrect dosing	11/28 (39)	1	*p* = 0.23	1	*p* = 0.05
Correct dosing	52/100 (52)	1.67 (0.71, 3.93)		2.52 (0.96, 6.58)	

*p*-values are log likelihood ratio tests; *CI*, confidence interval

^a^Three clients with incomplete data were excluded

^b^Adjusted for all factors in table except adherence factors denoted by superscript ^d^. Factors were not adjusted for adherence to avoid risk of over-adjustment bias given that adherence mediates viral load

^c^Factor modelled as a linear effect; *p(tr)* = *p*(trend)

^d^Incorrect dosing was determined by comparing self-reported dosing schedules to standard dosing schedules [45]

### Round Two

By the end of the *Round Two* follow-up period, 73% (116/159) of all *Round One* participants had been engaged by a specialist adherence counselor and 3% (4/159) were switched to second-line ART. Among clients recruited in *Round One*, 60% (44/73) of refugees and 58% (50/86) of host nationals submitted a blood sample in *Round Two*. There were no differences between those with or without a follow-up viral load in relation to refugee status (*p* = 0.96), age (*p* = 0.46) and gender (*p* = 0.23). However, among the group participating in *Round Two*, higher proportions were married (50% vs 30%, *p* = 0.02) and had not travelled for ≥1 consecutive month in the past year (30% vs 14%, *p* = 0.03) (Additional file 5). Among refugees and host nationals who did not participate in *Round Two*, a greater proportion of the host national group had travelled for ≥1 consecutive month in the past year (Table 1). Among those who participated in both rounds, median viral load declined between rounds (5,010 copies/mL vs 1,368, *p* < 0.002). The follow-up result was similar for refugees and host nationals (1,311 copies/mL vs 1,411, *p* = 0.41). Among all who tested <5000 copies/mL in *Round One*, 63% (24/38) maintained viral suppression in *Round Two* (71%, 10/14 refugees; 58%, 14/24 host nationals). Proportions of refugees and host nationals suppressed in *Round Two* were similar (74% vs 70%, *p* = 0.66) (Table 2).

**Fig. 2:**
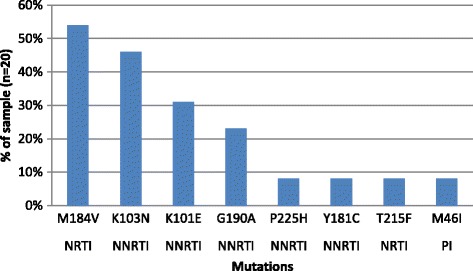
Prevalence of drug resistant mutations (*n* = 20). Legend: NRTI = nucleoside reverse transcriptase inhibitor; NNRTI = non-nucleoside reverse transcriptase inhibitor; PI = protease inhibitor. Mutations included M184V = 54% (7/13) of samples; K103N = 46% (6/13); K101E = 31% (4/13); G190A =23% (3/13); P225H = 8%; Y181C = 8%, T215F = 8%; M46I = 8% (1/13)

In samples submitted for drug resistance testing (*n* = 20; 8 refugees, 12 host nationals), 65% (13/20; 5 refugees, 8 host nationals) were successfully amplified. Of these, 69% (9/13) exhibited drug resistant mutations (4/5 refugees, 5/8 host nationals). Resistance to two drug classes was observed in 62% (8/13) of samples. The most prevalent mutations were M184V (NRTI-associated mutation) found in 54% (7/13) of samples and the K103N (NNRTI-associated mutation) found in 46% (6/13) of samples (Fig. 2). HIV-1 subtypes included sub-type A (62%; 8/13), C (15%; 2/13), and D (23%; 3/13).

## Discussion

In this study, the first of its kind to compare adherence to ART and viral suppression among refugees and host nationals who accessed ART from a refugee camp clinic, we found unacceptably low proportions of viral suppression within both groups. Among those on treatment for ≥25 weeks in the *Round One* survey, only 50% had a viral load of <5000 copies/mL. There was weak evidence that refugees had attained a better viral response when compared with host nationals in the *Round One* survey (*p* = 0.10); however, in the final multivariable model refugee status was not associated with viral suppression. Proportions suppressed within each group were also similar in *Round Two*.

Although improved in *Round Two*, proportions virologically suppressed continued to be unacceptably low in relation to other resource-limited settings [18–20]. Although virologic data from conflict-affected populations are sparse, a South African study found that foreigners had improved chances of viral suppression when compared with locals [21]. In Kuala Lumpur, Malaysia, high proportions of refugees and host nationals were virologically suppressed (81% v. 84%, *p* = 0.54) [6]. Strong outcomes among refugees as measured by survival, CD4 counts and adherence were found in other conflict-affected settings [22–24]. In the present study, the overall proportion suppressed was lower in comparison to findings from a meta-analysis of 89 studies of sub-Saharan African HIV treatment programs that reported 67% viral suppression after 12 months of ART [25].

In the present study, with the exception of host national self-reports over the past month, mean adherence in *Round One* using any measurement was >85%. When assessing these findings in relation to viral response and leaving the possibility of drug resistance aside, it is advisable that more sensitive measures of adherence be used for monitoring purposes, especially where laboratory monitoring is intermittent or unavailable. Notably, self-reported dosing, which was defined as an incorrect self-report of dosing schedule when compared with routine dosing schedules, was associated with lack of viral suppression. As ART tablets were distributed in small bags with non-standardized, handwritten dosing instructions, prescribed changes in the dosing or regimen may not have been understood. Improvements in *Round Two* outcomes suggested that basic program infrastructure, including supportive services, may have been lacking.

The effect of longer duration between HIV diagnosis and ART start on viral suppression (AOR: 3.98, 95% CI 1.44, 11.01; *p-trend* = 0.006) could have been related to effective patient monitoring that enabled better preparedness for ART when initiated in the home country or in the refugee camp setting. A shorter duration between diagnosis and initiation may signal a lack of engagement with health services. In other settings, delayed ART initiation was shown to be a barrier to retention in care [26, 27].

In drug resistance testing, 64% of successful amplifications exhibited resistance mutations. Assuming conservatively that the six unsuccessful amplifications tested negative, 45% of samples would still have exhibited major resistance mutations. This suggests that drug resistance may be a serious problem within this population. In 2011, eight years after ART roll-out in East Africa, the estimated prevalence of drug resistance among ART-naive individuals was 7.4% and was increasing by 29% per year [28]. In two contrasting Kenyan studies, the prevalence of transmitted resistance between 2008 and 2010 was 1.1% in a rural setting and 13.2% in an urban setting [29, 30]. Higher levels of drug resistance in our study population could have been linked to a history of treatment interruptions before, during, or after displacement, but prior to the self-reported recall period used to assess adherence in our survey. This could help to explain the discordance we detected between adherence and viral suppression, whereby proportions suppressed were much lower than what adherence levels would suggest. Early-warning indicators for drug resistance in African settings depend on prescribing practices, patient retention, continuity of drug supply, and viral suppression; therefore, effective patient monitoring is critical when drug resistance testing is not routinely available [31, 32].

Some important factors were not assessed in this study. These included the effects of very high viral loads at ART start, reduced potency of medications resulting from hot storage conditions, and acute malnutrition. Very high viral loads (≥100,000 copies/mL) are associated with a lower likelihood of ever achieving viral suppression [33]. Since viral load testing had not previously been implemented among this population, it was not possible to evaluate this possibility. In *Round One*, however, 15% (24/159) had a viral load in excess of 100,000 copies/mL. Although we did not measure acute malnutrition quantitatively, in accompanying qualitative work many clients reported that they were food insecure and believed that ART was toxic when taken without sufficient food [34]. Associations between food insecurity, viral load and mortality have been reported in other populations [35, 36] and are thought to be mediated by drug absorption and adherence [37–40]. Future studies conducted in refugee settings should use a sensitive measure of food insecurity to assess its true impact. Finally, we did not test the potency of medications, therefore any impact of storage conditions on potency could not be determined. The average annual temperature in Kakuma is 28 °C and can reach 40 °C or more in the dry season. The medications were stored in an indoor room that was not air conditioned.

This study had several limitations. Adherence self-reports may have suffered from recall and/or social desirability biases. As the host national community were primarily Turkana, a highly mobile group, findings may not be generalizable to other Kenyan host nationals. Bias may have been introduced to our comparisons of refugees and host nationals if a local clinic at Kakuma Mission Hospital had served healthier HIV-positive Kenyans. We could not assess this bias as we did not study this clinic. Risk factors were not assessed in *Round Two* due to a lack of resources to implement a follow-up survey questionnaire. Differences in mobility between refugees and host nationals may have introduced bias if one group had been less likely to participate in *Round Two* [41, 42]. If this had resulted in a lower likelihood of refilling prescriptions, for example, viral suppression could have been overestimated﻿ in one group﻿ compared with the other. The fact that few clients in *Round Two* had travelled for at least one consecutive month in the year prior to the study when compared with those who participated only in *Round One*, suggested that the *Round Two* cohort was more stable. Interestingly, the *Round Two* cohort included a greater proportion of host nationals who had travelled outside of the camp for one month or more in the year prior to the study, yet fewer were virologically suppressed in comparison to the refugee group. Although the overall *n* was small, a notable strength of the study was its relative completeness as 85% of all eligible clients were initially recruited. Strong efforts were made to mitigate recall and social desirability biases by conducting face-to-face interviews with well-trained local researchers. As Kakuma was founded in 1992, this setting was relatively stable and, arguably not generalizable to other acute humanitarian settings; however, recent but periodic influxes of refugees due to the intensification of conflicts in South Sudan and Somalia suggest that this stability was unreliable. Given that refugee settings around the world are often protracted, these findings might inform other, relatively stable refugee camp settings.

## Conclusions

In summary, unacceptably low proportions of refugees and host nationals were virologically suppressed in cross-sectional surveys conducted at two time-points. A remedial intervention that strengthened clinic procedures and adherence support between the surveys may have helped to improve outcomes; however, this finding was tentative given attrition between survey rounds. The proportions suppressed at <5000 copies/mL in *Round Two* were still unacceptably low, and drug resistance mutations were found in a high proportion of those who were not suppressed. Overall, there were only minor differences between refugees and host nationals across outcome measures, suggesting that socio-structural factors operating at the clinic level, not refugee status, were the best explanation for observed levels of viral suppression. Future work should evaluate interventions to improve adherence counseling, monitoring, and viral suppression across the range of settings where refugees and host nationals share HIV services, including acute and longer-term humanitarian settings and settings located outside of formal encampments. Within the framework of a public health approach to ART focused on increasing levels of viral suppression by retaining patients across the HIV cascade of care, these results have highlighted the importance of laboratory and counseling services for vulnerable populations who access services in remote settings. Given that early detection of treatment failures can prevent drug resistance [43] and consistent viral suppression limits onward HIV transmission [44], routine viral load testing using dried blood spots and/or point of care devices should be adopted in view of the potential long-term benefits. This study serves as a clear reminder that programs operating in remote settings should monitor performance carefully in an effort to maintain consistently high levels of viral suppression.

## Additional files

Additional file 1: Association of contextual factors with suppressed viral load among refugees and host community on ART for ≥25 weeks at baseline in Kakuma, Kenya (N=128^a^). (DOC 64 kb)

Additional file 2: Association of self-change factors with suppressed viral load among refugees and host community on ART for ≥25 weeks at baseline in Kakuma, Kenya (N=128^a^). (DOC 42 kb)

Additional file 3: Association of adherence measures (action state factors) with viral suppression among refugees and host nationals on ART for ≥25 weeks at baseline (*Round One*) in Kakuma, Kenya (N=128^a^). (DOC 38 kb)

Additional file 4: Mean adherence among refugees and host nationals at baseline (*Round One*) in Kakuma, Kenya. (DOCX 15 kb)

Additional file 5: Socio-demographic comparison of ART clients with and without a follow-up (*Round Two*) viral load in Kakuma, Kenya. (DOC 37 kb)

## Abbreviations

ART: antiretroviral therapy
IQR: interquartile range
IRC: International Rescue Committee
UNHCR: United Nations High Commissioner for Refugees
NRTI: nucleoside reverse transcriptase inhibitor
NNRTI: non-nucleoside reverse transcriptase inhibitor
WHO: World Health Organization
KEMRI: Kenya Medical Research Institute
CI: confidence interval
OR: odds ratio
AOR: adjusted odds ratio
PI: protease inhibitor
