# Supplementary material for: Low levels of viral suppression among refugees and host nationals accessing antiretroviral therapy in a Kenyan refugee camp
Source: Confl Health. 2017 Jun 2;11:11. doi: 10.1186/s13031-017-0111-3 (PMC5450054; doi:10.1186/s13031-017-0111-3)
Supplement: Supplementary file 1 — Association of contextual factors with suppressed viral load among refugees and host community on ART for ≥25 weeks at baseline in Kakuma, Kenya (N=128a). (DOC 64 kb) [file 13031_2017_111_MOESM1_ESM.doc]

**Additional file 1: Association of contextual factors with viral suppression among refugees and host nationals on ART for ≥25 weeks at baseline in Kakuma, Kenya (N=128^a^)**

| Factor | Prevalence <5000 copies/mL, n/N (%) | Crude odds ratio (95% CI) | *p*-value | Adjusted odds ratio (95% CI)^b^ | *p*-value |
| --- | --- | --- | --- | --- | --- |
| **Age group (years)** |  |  |  |  |  |
| 18+ | 14/31 (45) | 1 | *p(tr)*=0.90 | 1 | *p(tr)*=0.97 |
| 30+ | 33/63 (52) | 1.34 (0.56, 3.17) |  | 1.55 (0.60, 3.99) |  |
| 40+ | 16/34 (47) | 1.08 (0.41, 2.87) |  | 1.04 (0.35, 3.05) |  |
| **Time on ART (years)** |  |  |  |  |  |
| 0+ | 9/20 (45) | 1 | *p(tr)*=0.49 | 1 | *p(tr)*=0.69 |
| 1+ | 19/41 (46) | 1.06 (0.36, 3.09) |  | 0.72 (0.23, 2.28) |  |
| 3+ | 35/67 (52) | 1.34 (0.49, 3.64) |  | 0.75 (0.24, 2.29) |  |
| **Refugee status** |  |  |  |  |  |
| Kenyan | 30/71 (42) | 1 | *p*=0.08 | 1 | *p*=0.46 |
| Refugee | 33/57 (58) | 1.88 (0.93, 3.81) |  | 0.51 (0.08, 3.18) |  |
| **Gender** |  |  |  |  |  |
| Male | 20/44 (46) | 1 | *p*=0.54 | 1 | *p*=0.42 |
| Female | 43/84 (51) | 1.26 (0.61, 2.62) |  | 1.38 (0.62, 3.07) |  |
| **Travel for ≥1 continuous month, past year** |  |  |  |  |  |
| Travel | 10/26 (39) | 1 | *p*=0.22 | 1 | *p*=0.48 |
| No travel | 53/102 (52) | 1.73 (0.72, 4.17) |  | 1.41 (0.54, 3.63) |  |
| **Household size** |  |  |  |  |  |
| 1+ | 20/45 (44) | 1 | *p(tr)*=0.35 | 1 | *p(tr)*=0.37 |
| 5+ | 29/58 (50) | 1.25 (0.57, 2.73) |  | 1.33 (0.57, 3.14) |  |
| 9+ | 14/25 (56) | 1.59 (0.59, 4.26) |  | 1.60 (0.54, 4.73) |  |
| **Average time to clinic** **(hours)** |  |  |  |  |  |
| 1+ | 49/100 (49) | 1 | *p*=0.93 | 1 | *p*=0.43 |
| 0- | 14/28 (50) | 1.04 (0.45, 2.41) |  | 0.67 (0.25, 1.82) |  |
| **Personal income** |  |  |  |  |  |
| Any | 17/37 (46) | 1 | *p*=0.12 | 1 | *p*=0.93 |
| None | 46/91 (51) | 1.20 (0.56, 2.59) |  | 1.04 (0.45, 2.38) |  |
| **Time from HIV diagnosis to ART start (weeks)^c^** |  |  |  |  |  |
| 0- | 8/29 (28) | 1 | *p(tr)*=0.006 | 1 | *p(tr)*=0.006 |
| 24- | 6/13 (46) | 2.25 (0.58, 8.78) |  | 2.80 (0.66, 11.82) |  |
| 48+ | 49/86 (57) | 3.48 (1.39, 8.72) |  | 3.98 (1.44, 11.01) |  |
| **Place of ART start** |  |  |  |  |  |
| Kenya | 32/78 (41) | 1 | *p*=0.02 | 1 | *p*=0.13 |
| Elsewhere | 31/50 (62) | 2.35 (1.13, 4.86) |  | 3.98 (0.62, 25.55) |  |
| **ART regimen** |  |  |  |  |  |
| Nevirapine-based | 46/97 (47) | 1 | *p*=0.47 | 1 | *p*=0.53 |
| Efavirenz-based | 17/31 (55) | 1.35 (0.60, 3.03) |  | 1.33 (0.55, 3.19) |  |
| **Symptom or side-effect, past four weeks** |  |  |  |  |  |
| No symptoms/side-effect | 50/102 (49) | 1 | *p*=0.93 | 1 | *p*=0.87 |
| Any symptoms/side-effect | 13/26 (50) | 1.04 (0.44, 2.46) |  | 0.93 (0.37, 2.35) |  |
| **Regimen change, ever** |  |  |  |  |  |
| Previous change | 37/69 (54) | 1 | *p*=0.28 | 1 | *p*=0.26 |
| No change | 26/59 (44) | 1.47 (0.73, 2.95) |  | 1.55 (0.72, 3.35) |  |
| **Prescription refill difficulties, past three months** |  |  |  |  |  |
| No difficulties | 53/111 (48) | 1 | *p*=0.39 | 1 | *p*=0.13 |
| Difficulties | 10/17 (59) | 1.56 (0.56, 4.40) |  | 2.43 (0.75, 7.92) |  |
| **Food security ^d^** |  |  |  |  |  |
| Food insecure | 45/96 (47) | 1 | *p*=0.36 | 1 | *p*=0.18 |
| Food secure | 18/32 (56) | 1.46 (0.65, 3.26) |  | 1.80 (0.76, 4.27) |  |
| *p*-values are log likelihood ratio tests; CI=confidence interval  ^a^ Three clients with incomplete data were excluded  ^b^ Adjusted for age group, refugee status, time on ART, time from HIV diagnosis to ART start, place of ART start, refill difficulties, food security  ^c^ Factor modelled as a linear effect; *p(tr)*=*p*(trend)  ^d^ Constructed from three questions, each measured on a 3-point Likert scale. An endorsement of “some of the time” or “ all of the time” on any of the three questions was scored as “insecure” | | | | | |
