# Supplementary material for: Low levels of viral suppression among refugees and host nationals accessing antiretroviral therapy in a Kenyan refugee camp
Source: Confl Health. 2017 Jun 2;11:11. doi: 10.1186/s13031-017-0111-3 (PMC5450054; doi:10.1186/s13031-017-0111-3)
Supplement: Supplementary file 2 — Association of self-change factors with suppressed viral load among refugees and host community on ART for ≥25 weeks at baseline in Kakuma, Kenya (N=128a). (DOC 42 kb) [file 13031_2017_111_MOESM2_ESM.doc]

Additional file 2: Association of self-change factors with viral suppression among refugees and host nationals on ART for ≥25 weeks at baseline *(Round One)* in Kakuma, Kenya (N=128^a^)

| Factor | Prevalence <5000 copies/mL, n/N (%) | Crude odds ratio (95% CI) | *p*-value | Adjusted odds ratio (95% CI)^b^ | *p*-value |
| --- | --- | --- | --- | --- | --- |
| **Adherence self-efficacy (self-reported ability to take medications as prescribed over previous month)** |  |  |  |  |  |
| Excellent/very good | 59/68 (87) | 1 | *p*=0.85 | 1 | *p*=0.40 |
| Good/fair/poor/very poor | 54/60 (90) | 1.07 (0.53, 2.14) |  | 0.70 (0.30, 1.62) |  |
| **Serostatus disclosure to partner** |  |  |  |  |  |
| Serostatus disclosed | 32 /75 (43) | 1 | *p*=0.09 | 1 | *p*=0.24 |
| Serostatus undisclosed | 17/25 (68) | 2.86 (1.10, 7.43) |  | 2.51 (0.82, 7.71) |  |
| No partner | 14/28 (50) | 1.34 (0.56, 3.21) |  | 1.39 (0.52, 3.72) |  |
| **Serostatus disclosure to non-partner others** |  |  |  |  |  |
| Serostatus undisclosed | 48/100 (48) | 1 | *p*=0.60 | 1 | *p*=0.71 |
| Serostatus disclosed | 15/28 (54) | 1.25 (0.54, 2.90) |  | 1.20 (0.47, 3.09) |  |
| **Alcohol use, past month** |  |  |  |  |  |
| 1+ times | 5/16 (31) | 1 | *p*=0.12 | 1 | *p*=0.15 |
| 0 times | 58/112 (52) | 2.36 (0.77, 7.24) |  | 2.38 (0.70, 8.03) |  |
| **No. of reported barriers to adherence^de^** |  |  |  |  |  |
| 5+ | 16/37 (43) | 1 | *p(tr)*=0.37 | 1 | *p(tr)*=0.82 |
| 2+ | 21/42 (50) | 1.31 (0.54, 3.19) |  | 1.27 (0.47, 3.41) |  |
| 0+ | 26/49 (53) | 1.48 (0.63, 3.50) |  | 1.03 (0.34, 2.44) |  |
| **Knowledge of HIV and AIDS (#correct/4)** |  |  |  |  |  |
| 100% | 43/90 (48) | 1 | *p*=0.62 | 1 | *p*=0.70 |
| <100% | 20/38 (53) | 1.21 (0.57, 2.60) |  | 1.19 (0.50, 2.82) |  |
| *p*-values are log likelihood ratio tests; CI=confidence interval  ^a^ Three clients with incomplete data were excluded  ^b^ Adjusted for age group, refugee status, time on ART, time from HIV diagnosis to ART start, place of ART start, refill difficulties, food security, alcohol use  ^c^ Examples of illegal or harmful substances were suggested by interviewers (e.g., Khat, marijuana), but defined by clients  ^d^ Factor modelled as a linear effect; *p(tr)*=*p*(trend)  ^e^ Potential barriers included: being away from home; busy with other tasks; too many pills; avoidance of side-effects; hide medication taking from others; change in daily routine; perception of drug as toxic or harmful; fell asleep during dosing time; illness; depression; problems keeping to dosing schedule; run out of medication; detained or incarcerated; irritability/angry; less interest in daily activities; less skills; difficulty dealing with new situations; unable to make daily plans; worry; hopelessness; suspicious of treatment; want to be free of medicines; financial constraints; decreased quality of life; uncertainty; others self-reported | | | | | |
