# Supplementary material for: Low levels of viral suppression among refugees and host nationals accessing antiretroviral therapy in a Kenyan refugee camp
Source: Confl Health. 2017 Jun 2;11:11. doi: 10.1186/s13031-017-0111-3 (PMC5450054; doi:10.1186/s13031-017-0111-3)
Supplement: Supplementary file 3 — Association of adherence measures (action state factors) with viral suppression among refugees and host nationals on ART for ≥25 weeks at baseline (Round One) in Kakuma, Kenya (N=128a). (DOC 38 kb) [file 13031_2017_111_MOESM3_ESM.doc]

Additional file 3: Association of adherence measures (action state factors) with viral suppression among refugees and host nationals on ART for ≥25 weeks at baseline *(Round One)* in Kakuma, Kenya (N=128^a^)

| Factor | Prevalence <5000 copies/mL, n/N (%) or mean (95%CI) | Crude odds ratio (95% CI) | *p*-value | Adjusted odds ratio (95% CI)^b^ | *p*-value |
| --- | --- | --- | --- | --- | --- |
| **Adherence to medication schedule, self-reported** |  |  |  |  |  |
| Inconsistent | 7/25 (28) | 1 | *p*=0.02 | 1 | *p*=0.04 |
| Consistent | 56/103 (54) | 3.06 (1.18, 7.96) |  | 2.80 (0.99, 7.90) |  |
| **Adherence, visual analogue scale self-report, past month (by 5% intervals)** | 89.74 (87.04, 92.44) | 1.12 (0.96, 1.30) | *p*=0.11 | 0.71 (0.44, 1.14) | *p*=0.05 |
| **Adherence, dose-by-dose self-report, past four days (by 5% intervals )** | 93.36 (89.46, 97.26) | 1.04 (0.95, 1.14) | *p*=0.38 | 0.99 (0.86, 1.15) | *p*=0.94 |
| **Adherence to pharmacy refill schedule (by 5% intervals)** | 92.87 (89.78, 95.95) | 1.02 (0.91, 1.14) | *p*=0.72 | 1.01 (0.86, 1.19) | *p*=0.91 |
| **Treatment interruptions of ≥2 days, self-report, past six months** |  |  |  |  |  |
| Any reported interruption | 53/101 (53) | 1 | *p*=0.15 | 1 | *p*=0.13 |
| No reported interruption | 10/27 (37) | 1.88 (0.78, 4.50) |  | 0.45 (0.16, 1.29) |  |
| **Self-reported dosing schedule ^c^** |  |  |  |  |  |
| Incorrect dosing | 11/28 (39) | 1 | *p*=0.23 | 1 | *p*=0.02 |
| Correct dosing | 52/100 (52) | 1.67 (0.71, 3.93) |  | 3.33 (1.20, 9.24) |  |
| *p*-values are log likelihood ratio tests; CI=confidence interval  ^a^ Three clients with incomplete data were excluded  ^b^ Adjusted for age group, refugee status, time on ART, time from HIV diagnosis to ART start, place of ART start, refill difficulties, food security, alcohol use  ^c^ Incorrect dosing was determined by comparing self-reported dosing schedules to standard dosing schedules | | | | | |
