# Supplementary material for: Low levels of viral suppression among refugees and host nationals accessing antiretroviral therapy in a Kenyan refugee camp
Source: Confl Health. 2017 Jun 2;11:11. doi: 10.1186/s13031-017-0111-3 (PMC5450054; doi:10.1186/s13031-017-0111-3)
Supplement: Supplementary file 4 — Mean adherence among refugees and host nationals at baseline (Round One) in Kakuma, Kenya. (DOCX 15 kb) [file 13031_2017_111_MOESM4_ESM.docx]

Additional file 4: Mean adherence among refugees and host nationals at baseline *(Round One)* in Kakuma, Kenya

|  | | | | | | | | |
| --- | --- | --- | --- | --- | --- | --- | --- | --- |
|  | On treatment, ≥25 weeks | | | | On treatment, all | | | |
| Measure (mean %, CI) | All (n=131) | Refugee (n=59) | Kenyan (n=72) | *p*-value | All (n=159) | Refugee (n=73) | Kenyan (n=86) | *p*-value |
| Four-day self-report, dose by dose | 93.1 (89.2, 97.0) ^a^ | 94.2 (88.8, 99.5) ^b^ | 92.2 (86.5, 97.9) | 0.62 | 92.8 (89.1, 96.4) ^c^ | 93.3 (88.2, 98.4) | 92.3 (87.1, 97.5) | 0.78 |
| One month self-report, visual analog scale | 88.9 (85.9, 91.9) | 90.8 (86.2, 95.3) | 87.3 (83.4, 91.3) | 0.26 | 88.5 (85.7, 91.2) | 90.1 (85.9, 94.3) | 87.1 (83.4, 90.7) | 0.28 |
| Pharmacy refill schedule, 24 months | 92.9 (89.9, 95.9) | 95.6 (91.6, 99.6) | 90.7 (91.6, 99.6) | 0.11 | 93.1 (90.2, 95.9) | 94.1 (89.6, 98.6) | 92.2 (88.4, 95.9) | 0.51 |
| *p*-values are t-tests; CI=confidence interval  ^a^ n=130; ^b^ n=58; ^c^ n=158 | | | | | | | | |
