# Supplementary material for: Low levels of viral suppression among refugees and host nationals accessing antiretroviral therapy in a Kenyan refugee camp
Source: Confl Health. 2017 Jun 2;11:11. doi: 10.1186/s13031-017-0111-3 (PMC5450054; doi:10.1186/s13031-017-0111-3)
Supplement: Supplementary file 5 — Socio-demographic comparison of ART clients with and without a follow-up (Round Two) viral load in Kakuma, Kenya. (DOC 37 kb) [file 13031_2017_111_MOESM5_ESM.doc]

Additional file 5: Socio-demographic comparison of ART clients with and without a follow-up *(Round Two)* viral load in Kakuma, Kenya

| Factor | Not included in Round Two (n=53) | Remaining in Round Two (n=78) | *p*-value |
| --- | --- | --- | --- |
| **Age, median yrs (IQR)** | 35 (30, 43) | 34 (30, 39) | *p*=0.46^a^ |
| **Refugee status** | 24 (45) | 35 (45) | *p*=0.96 |
| **Female gender** | 38 (72) | 48 (62) | *p*=0.23 |
| **No earned income ^c^** | 45 (85) | 61 (78) | *p*=0.34 |
| **Married/cohabiting** | 16 (30) | 39 (50) | *p*=0.02 |
| **Nationality** |  |  | *p*=0.26^b^ |
| Kenyan | 29 (55) | 43 (55) |  |
| Somali, Ethiopian, Eritrean ^d^ | 15 (28) | 14 (18) |  |
| Sudanese | 4 (8) | 14 (18) |  |
| Rwandese, Congolese, Burundian | 5 (9) | 7 (9) |  |
| **Travel for ≥1 continuous month in past year** | 16 (30) | 11 (14) | *p*=0.03 |
| **Self-reported consistent adherence to medication schedule** | 40 (77) | 64 (82) | *p*=0.47 |
| **Incorrect ART dosing** | 11 (21) | 17 (22) | *p*=0.89 |
| **Time on ART, median wks (IQR)** | 148 (82, 252) | 190 (105, 257) | *p*=0.62^a^ |
| **Time from HIV diagnosis to ART, median wks (IQR)** | 12 (0, 47) | 3 (0, 43) | *p*=0.18^a^ |
| **Time in host country, median wks (IQR)** | 506 (234, 809) | 596 (309, 948) | *p*=0.40^a^ |
| Values are numbers (%) unless otherwise stated; *p*-values are chi-square tests unless otherwise stated; IQR=interquartile range  ^a^ Mann-Whitney two-sample statistic (Wilcoxon rank-sum test); ^b^ Fisher’s exact test  ^c^ Not including financial assistance provided within refugee camp  ^e^ Somalis, Ethiopians, and Eritreans were grouped together as Somalis often reported Ethiopian nationality to conceal identities | | | |
